# 2-(2-Oxo-2-phenyl­ethyl)-1,2-benziso­thia­zol-3(2*H*)-one 1,1-dioxide

**DOI:** 10.1107/S1600536810005404

**Published:** 2010-02-13

**Authors:** Matloob Ahmad, Hamid Latif Siddiqui, Muhammad Azam, Iftikhar Hussain Bukhari, Masood Parvez

**Affiliations:** a Institute of Chemistry, University of the Punjab, Lahore, Pakistan; b Institute of Biochemistry, University of Baluchistan, Quetta 8700, Pakistan; c Department of Chemistry, University of Sargodha, Sargodha 10400, Pakistan; d Department of Chemistry, The University of Calgary, 2500 University Drive NW, Calgary, Alberta, Canada T2N 1N4

## Abstract

In the title compound, C_15_H_11_NO_4_S, the benzothia­zole unit is essentially planar [maximum deviation = 0.0644 (14) Å for the N atom] and forms a dihedral angle 54.43 (6)° with the phenyl ring. In the crystal structure, weak bifurcated C—H⋯O hydrogen bonds involving the carbonyl O atoms as acceptors result in *R*_2_^2^(7) ring motifs.

## Related literature

For the use of 1,2-benzisothia­zoline-3-one 1,1-dioxide (saccharine) as an inter­mediate in the preparation of medicinally important mol­ecules, see: Siddiqui *et al.* (2006 ▶); Zia-ur-Rehman *et al.* (2005 ▶, 2009 ▶). For the biological activity of saccharine, see: Singh *et al.* (2007 ▶); Vaccarino *et al.* (2007 ▶); Kapui *et al.* (2003 ▶). For related structures, see: Ahmad *et al.* (2008 ▶, 2009 ▶). For hydrogen-bonding motifs, see: Bernstein *et al.* (1995 ▶).
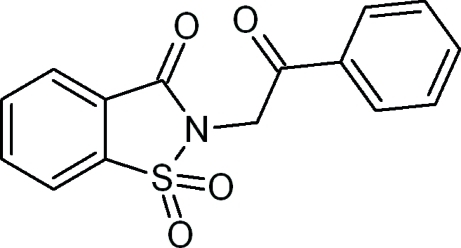


## Experimental

### 

#### Crystal data

C_15_H_11_NO_4_S
*M* *_r_* = 301.31
Monoclinic, 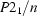

*a* = 8.0730 (2) Å
*b* = 9.1270 (3) Å
*c* = 18.0143 (6) Å
β = 95.4616 (18)°
*V* = 1321.31 (7) Å^3^
*Z* = 4
Mo *K*α radiation
μ = 0.26 mm^−1^
*T* = 173 K
0.18 × 0.14 × 0.10 mm

#### Data collection

Nonius diffractometer with Bruker APEXII CCD
Absorption correction: multi-scan (*SORTAV*;Blessing, 1997 ▶) *T*_min_ = 0.955, *T*_max_ = 0.974
15562 measured reflections
3016 independent reflections
2554 reflections with (*I*) > 2.0 σ(*I*)
*R*_int_ = 0.030

#### Refinement

*R*[*F* ^2^ > 2σ(*F* ^2^)] = 0.045
*wR*(*F* ^2^) = 0.115
*S* = 1.09
3016 reflections
190 parameters
H-atom parameters constrained
Δρ_max_ = 0.31 e Å^−3^
Δρ_min_ = −0.43 e Å^−3^

### 

Data collection: *COLLECT* (Hooft, 1998 ▶); cell refinement: *HKL* *DENZO* (Otwinowski & Minor, 1997 ▶); data reduction: *SCALEPACK* (Otwinowski & Minor, 1997 ▶); program(s) used to solve structure: *SHELXS97* (Sheldrick, 2008 ▶); program(s) used to refine structure: *SHELXL97* (Sheldrick, 2008 ▶); molecular graphics: *ORTEP-3 for Windows* (Farrugia, 1997 ▶); software used to prepare material for publication: *SHELXL97*.

## Supplementary Material

Crystal structure: contains datablocks Global, I. DOI: 10.1107/S1600536810005404/lh2990sup1.cif

Structure factors: contains datablocks I. DOI: 10.1107/S1600536810005404/lh2990Isup2.hkl

Additional supplementary materials:  crystallographic information; 3D view; checkCIF report

## Figures and Tables

**Table 1:** Hydrogen-bond geometry (Å, °)

*D*—H⋯*A*	*D*—H	H⋯*A*	*D*⋯*A*	*D*—H⋯*A*
C8—H8*A*⋯O1^i^	0.99	2.41	3.318 (3)	153
C15—H15⋯O1^i^	0.95	2.47	3.417 (3)	178

Symmetry code: (i) 
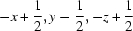
.

**Fig. 1.**
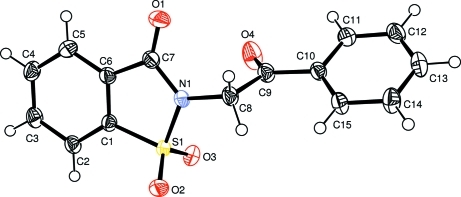
ORTEP-3 (Farrugia, 1997) drawing of (I) with displacement ellipsoids plotted at 50% probability level.

**Fig. 2.**
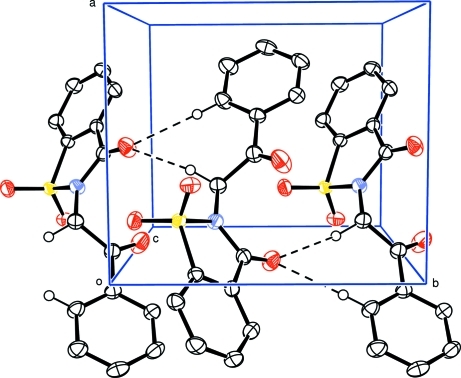
Unit cell packing of (I) showing non-classical hydrogen bonding interaction with dashed lines; H-atoms not involved in H-bonds have been excluded for clarity.

## supplementary crystallographic information

### Comment

1,2-Benzisothiazoline-3-one 1,1-dioxide (saccharine) is an important starting material for the synthesis of different heterocyclic compounds and plays a role as an intermediate for the preparation of medicinally important molecules (Siddiqui *et al.*, 2006; Zia-ur-Rehman *et al.*, 2009). Various derivatives of saccharin are known to be cyclooxygenase-2 (COX-2) inhibitors (Singh *et al.*, 2007), analgesic (Vaccarino *et al.*, 2007), human leucocyte elastase (HLE) inhibitors (Kapui *et al.*, 2003) etc. In continuation of our research on the synthesis of potential biologically active derivatives of benzothiazines (Ahmad *et al.*, 2008; Ahmad *et al.*, 2009), we herein report the crystal structure of the title compound, *N*-phenacylsaccharin, (I).

The structure of (I) contains discrete molecules separated by normal van der Waals distances (Fig. 1). The benzothiazole moiety (S1/N1/C1—C7) is essentially planar (maximum deviation = 0.0644 (14) Å for N1-atom) and lies at an angle 54.43 (6) ° with respect to the phenyl ring (C10—C15). The structure is devoid of any classical hydrogen bonds. However, non-classical hydrogen bonding interactions of the type C—H···O are present in the crystal structure involving O1 and H-atoms bonded to C8 and C15 resulting in a seven membered ring in R_2_^2^(7) motif (Bernstein *et al.*, 1995) (Fig. 2 and Table 1).

### Experimental

Phenacyl bromide (4.85 g, 0.024 mol) was slowly added to a suspension of sodium saccharine (5 g, 0.024 mol) in dimethylformamide (15 ml) and the mixture was stirred at 383 K for 3 hours under anhydrous conditions. On completion of reaction (as indicated by tlc), the mixture was poured on crushed ice and the precipitates formed were filtered and washed with excess of distilled water and cold ethanol respectively. The crystals of *N*-phenacylsaccharin suitable for XRD were grown from a solution of chloroform-methanol (3:1).

### Refinement

All the H-atoms were located from the difference Fourier maps and were included in the refinements at geometrically idealized positions with C—H distances = 0.95 and 0.99 Å for aryl and methylene H-atoms, respectively, and U_iso_ = 1.2 times U_eq_ of the C-atoms to which they were bonded. The final difference map was free of chemically significant features.

### Crystal data

**Table:**

C_15_H_11_NO_4_S	*F*(000) = 624
*M**_r_* = 301.31	*D*_x_ = 1.515 Mg m^−^^3^
Monoclinic, *P*2_1_/*n*	Melting point: 458 K
Hall symbol: -P 2yn	Mo *K*α radiation, λ = 0.71073 Å
*a* = 8.0730 (2) Å	Cell parameters from 3115 reflections
*b* = 9.1270 (3) Å	θ = 1.0–27.5°
*c* = 18.0143 (6) Å	µ = 0.26 mm^−^^1^
β = 95.4616 (18)°	*T* = 173 K
*V* = 1321.31 (7) Å^3^	Block, white
*Z* = 4	0.18 × 0.14 × 0.10 mm

### Data collection

**Table:**

Nonius APEX2 CCD diffractometer	3016 independent reflections
Radiation source: fine-focus sealed tube	2554 reflections with (*I*) > 2.0 σ(*I*)
graphite	*R*_int_ = 0.030
φ & ω scans	θ_max_ = 27.5°, θ_min_ = 2.5°
Absorption correction: multi-scan (*SORTAV*;Blessing, 1997)	*h* = −10→10
*T*_min_ = 0.955, *T*_max_ = 0.974	*k* = −11→11
15562 measured reflections	*l* = −23→23

### Refinement

**Table:**

Refinement on *F*^2^	Primary atom site location: structure-invariant direct methods
Least-squares matrix: full	Secondary atom site location: difference Fourier map
*R*[*F*^2^ > 2σ(*F*^2^)] = 0.045	Hydrogen site location: inferred from neighbouring sites
*wR*(*F*^2^) = 0.115	H-atom parameters constrained
*S* = 1.09	*w* = 1/[σ^2^(*F*_o_^2^) + (0.0412*P*)^2^ + 1.31*P*] where *P* = (*F*_o_^2^ + 2*F*_c_^2^)/3
3016 reflections	(Δ/σ)_max_ < 0.001
190 parameters	Δρ_max_ = 0.31 e Å^−^^3^
0 restraints	Δρ_min_ = −0.43 e Å^−^^3^

### Special details

**Table:**

Geometry. All esds (except the esd in the dihedral angle between two l.s. planes) are estimated using the full covariance matrix. The cell esds are taken into account individually in the estimation of esds in distances, angles and torsion angles; correlations between esds in cell parameters are only used when they are defined by crystal symmetry. An approximate (isotropic) treatment of cell esds is used for estimating esds involving l.s. planes.
Refinement. Refinement of *F*^2^ against ALL reflections. The weighted *R*-factor wR and goodness of fit *S* are based on *F*^2^, conventional *R*-factors *R* are based on *F*, with *F* set to zero for negative *F*^2^. The threshold expression of *F*^2^ > σ(*F*^2^) is used only for calculating *R*-factors(gt) etc. and is not relevant to the choice of reflections for refinement. *R*-factors based on *F*^2^ are statistically about twice as large as those based on *F*, and *R*- factors based on ALL data will be even larger.

### Fractional atomic coordinates and isotropic or equivalent isotropic displacement parameters (Å^2^)

**Table:**

	*x*	*y*	*z*	*U*_iso_*/*U*_eq_	
S1	0.21297 (6)	0.21225 (5)	0.09588 (3)	0.02212 (14)	
O1	0.0329 (2)	0.51326 (18)	0.20418 (9)	0.0369 (4)	
O2	0.20831 (19)	0.06413 (16)	0.12149 (9)	0.0303 (4)	
O3	0.35091 (18)	0.25411 (18)	0.05634 (8)	0.0306 (4)	
O4	0.4233 (2)	0.5471 (2)	0.17286 (10)	0.0480 (5)	
N1	0.1956 (2)	0.3264 (2)	0.16706 (9)	0.0256 (4)	
C1	0.0221 (2)	0.2691 (2)	0.05015 (11)	0.0220 (4)	
C2	−0.0584 (3)	0.2109 (2)	−0.01446 (11)	0.0258 (4)	
H2	−0.0133	0.1309	−0.0396	0.031*	
C3	−0.2090 (3)	0.2757 (3)	−0.04073 (12)	0.0301 (5)	
H3	−0.2680	0.2395	−0.0851	0.036*	
C4	−0.2743 (3)	0.3918 (3)	−0.00347 (12)	0.0306 (5)	
H4	−0.3765	0.4347	−0.0231	0.037*	
C5	−0.1931 (3)	0.4467 (2)	0.06218 (12)	0.0292 (5)	
H5	−0.2394	0.5250	0.0882	0.035*	
C6	−0.0424 (2)	0.3840 (2)	0.08864 (11)	0.0239 (4)	
C7	0.0608 (3)	0.4209 (2)	0.15913 (11)	0.0251 (4)	
C8	0.3232 (3)	0.3329 (2)	0.22988 (11)	0.0264 (4)	
H8A	0.3841	0.2387	0.2336	0.032*	
H8B	0.2690	0.3462	0.2765	0.032*	
C9	0.4470 (3)	0.4575 (2)	0.22252 (11)	0.0274 (4)	
C10	0.5913 (2)	0.4682 (2)	0.28032 (11)	0.0242 (4)	
C11	0.6695 (3)	0.6038 (2)	0.29126 (13)	0.0294 (5)	
H11	0.6312	0.6856	0.2619	0.035*	
C12	0.8030 (3)	0.6194 (3)	0.34485 (13)	0.0333 (5)	
H12	0.8551	0.7122	0.3526	0.040*	
C13	0.8607 (3)	0.5001 (3)	0.38712 (12)	0.0347 (5)	
H13	0.9522	0.5114	0.4239	0.042*	
C14	0.7858 (3)	0.3650 (3)	0.37606 (13)	0.0354 (5)	
H14	0.8271	0.2828	0.4045	0.042*	
C15	0.6494 (3)	0.3493 (3)	0.32304 (12)	0.0291 (5)	
H15	0.5961	0.2569	0.3162	0.035*	

### Atomic displacement parameters (Å^2^)

**Table:**

	*U*^11^	*U*^22^	*U*^33^	*U*^12^	*U*^13^	*U*^23^
S1	0.0205 (2)	0.0219 (3)	0.0228 (2)	0.00177 (18)	−0.00385 (18)	−0.00180 (19)
O1	0.0420 (9)	0.0322 (9)	0.0349 (9)	0.0078 (7)	−0.0040 (7)	−0.0128 (7)
O2	0.0332 (8)	0.0218 (8)	0.0347 (8)	0.0030 (6)	−0.0036 (6)	0.0007 (6)
O3	0.0222 (7)	0.0374 (9)	0.0320 (8)	0.0010 (6)	0.0007 (6)	0.0023 (7)
O4	0.0550 (11)	0.0412 (10)	0.0430 (10)	−0.0156 (9)	−0.0195 (8)	0.0180 (8)
N1	0.0260 (9)	0.0255 (9)	0.0235 (8)	0.0023 (7)	−0.0075 (7)	−0.0049 (7)
C1	0.0206 (9)	0.0222 (10)	0.0227 (9)	−0.0007 (8)	−0.0009 (7)	0.0020 (8)
C2	0.0265 (10)	0.0274 (11)	0.0228 (10)	−0.0021 (8)	−0.0019 (8)	−0.0020 (8)
C3	0.0295 (11)	0.0353 (12)	0.0236 (10)	−0.0031 (9)	−0.0075 (8)	0.0018 (9)
C4	0.0248 (10)	0.0322 (12)	0.0333 (11)	0.0029 (9)	−0.0054 (9)	0.0044 (9)
C5	0.0282 (10)	0.0253 (11)	0.0335 (11)	0.0052 (9)	−0.0006 (9)	0.0008 (9)
C6	0.0247 (10)	0.0202 (10)	0.0261 (10)	−0.0009 (8)	−0.0011 (8)	0.0012 (8)
C7	0.0263 (10)	0.0216 (10)	0.0266 (10)	−0.0013 (8)	−0.0020 (8)	−0.0004 (8)
C8	0.0281 (10)	0.0274 (11)	0.0219 (10)	−0.0009 (8)	−0.0071 (8)	0.0000 (8)
C9	0.0295 (10)	0.0271 (11)	0.0242 (10)	−0.0015 (9)	−0.0039 (8)	0.0003 (8)
C10	0.0232 (9)	0.0282 (11)	0.0209 (9)	−0.0019 (8)	0.0002 (7)	−0.0039 (8)
C11	0.0261 (10)	0.0275 (11)	0.0344 (12)	−0.0016 (9)	0.0016 (9)	−0.0025 (9)
C12	0.0274 (11)	0.0335 (12)	0.0390 (13)	−0.0090 (9)	0.0031 (9)	−0.0088 (10)
C13	0.0260 (11)	0.0488 (15)	0.0282 (11)	−0.0086 (10)	−0.0031 (9)	−0.0044 (10)
C14	0.0320 (12)	0.0400 (13)	0.0321 (12)	−0.0056 (10)	−0.0079 (9)	0.0073 (10)
C15	0.0303 (11)	0.0304 (11)	0.0251 (10)	−0.0070 (9)	−0.0050 (8)	0.0013 (9)

### Geometric parameters (Å, °)

**Table:**

S1—O3	1.4298 (15)	C5—H5	0.9500
S1—O2	1.4301 (15)	C6—C7	1.489 (3)
S1—N1	1.6685 (18)	C8—C9	1.528 (3)
S1—C1	1.755 (2)	C8—H8A	0.9900
O1—C7	1.206 (3)	C8—H8B	0.9900
O4—C9	1.213 (3)	C9—C10	1.490 (3)
N1—C7	1.385 (3)	C10—C15	1.386 (3)
N1—C8	1.457 (2)	C10—C11	1.395 (3)
C1—C2	1.384 (3)	C11—C12	1.384 (3)
C1—C6	1.386 (3)	C11—H11	0.9500
C2—C3	1.394 (3)	C12—C13	1.383 (3)
C2—H2	0.9500	C12—H12	0.9500
C3—C4	1.386 (3)	C13—C14	1.380 (3)
C3—H3	0.9500	C13—H13	0.9500
C4—C5	1.390 (3)	C14—C15	1.395 (3)
C4—H4	0.9500	C14—H14	0.9500
C5—C6	1.387 (3)	C15—H15	0.9500
			
O3—S1—O2	117.32 (10)	N1—C7—C6	108.51 (17)
O3—S1—N1	110.03 (9)	N1—C8—C9	112.50 (17)
O2—S1—N1	109.61 (9)	N1—C8—H8A	109.1
O3—S1—C1	112.19 (9)	C9—C8—H8A	109.1
O2—S1—C1	112.48 (9)	N1—C8—H8B	109.1
N1—S1—C1	92.39 (9)	C9—C8—H8B	109.1
C7—N1—C8	123.03 (17)	H8A—C8—H8B	107.8
C7—N1—S1	115.60 (14)	O4—C9—C10	122.0 (2)
C8—N1—S1	121.16 (14)	O4—C9—C8	120.45 (19)
C2—C1—C6	122.85 (19)	C10—C9—C8	117.53 (17)
C2—C1—S1	127.02 (16)	C15—C10—C11	119.42 (19)
C6—C1—S1	110.12 (14)	C15—C10—C9	122.54 (19)
C1—C2—C3	116.6 (2)	C11—C10—C9	118.04 (19)
C1—C2—H2	121.7	C12—C11—C10	120.1 (2)
C3—C2—H2	121.7	C12—C11—H11	119.9
C4—C3—C2	121.4 (2)	C10—C11—H11	119.9
C4—C3—H3	119.3	C13—C12—C11	120.1 (2)
C2—C3—H3	119.3	C13—C12—H12	119.9
C3—C4—C5	121.1 (2)	C11—C12—H12	119.9
C3—C4—H4	119.4	C14—C13—C12	120.3 (2)
C5—C4—H4	119.4	C14—C13—H13	119.9
C6—C5—C4	118.1 (2)	C12—C13—H13	119.9
C6—C5—H5	120.9	C13—C14—C15	119.8 (2)
C4—C5—H5	120.9	C13—C14—H14	120.1
C1—C6—C5	119.97 (19)	C15—C14—H14	120.1
C1—C6—C7	113.03 (17)	C10—C15—C14	120.2 (2)
C5—C6—C7	126.91 (19)	C10—C15—H15	119.9
O1—C7—N1	124.10 (19)	C14—C15—H15	119.9
O1—C7—C6	127.35 (19)		
			
O3—S1—N1—C7	109.01 (16)	S1—N1—C7—O1	−177.54 (18)
O2—S1—N1—C7	−120.57 (16)	C8—N1—C7—C6	179.40 (18)
C1—S1—N1—C7	−5.67 (17)	S1—N1—C7—C6	4.5 (2)
O3—S1—N1—C8	−65.95 (18)	C1—C6—C7—O1	−178.4 (2)
O2—S1—N1—C8	64.47 (18)	C5—C6—C7—O1	−1.8 (4)
C1—S1—N1—C8	179.38 (17)	C1—C6—C7—N1	−0.5 (2)
O3—S1—C1—C2	72.9 (2)	C5—C6—C7—N1	176.0 (2)
O2—S1—C1—C2	−62.0 (2)	C7—N1—C8—C9	−77.6 (3)
N1—S1—C1—C2	−174.4 (2)	S1—N1—C8—C9	97.0 (2)
O3—S1—C1—C6	−107.75 (16)	N1—C8—C9—O4	7.7 (3)
O2—S1—C1—C6	117.42 (15)	N1—C8—C9—C10	−175.22 (18)
N1—S1—C1—C6	5.03 (16)	O4—C9—C10—C15	−160.3 (2)
C6—C1—C2—C3	1.2 (3)	C8—C9—C10—C15	22.7 (3)
S1—C1—C2—C3	−179.45 (16)	O4—C9—C10—C11	19.8 (3)
C1—C2—C3—C4	−0.4 (3)	C8—C9—C10—C11	−157.27 (19)
C2—C3—C4—C5	−0.9 (3)	C15—C10—C11—C12	−0.6 (3)
C3—C4—C5—C6	1.4 (3)	C9—C10—C11—C12	179.3 (2)
C2—C1—C6—C5	−0.8 (3)	C10—C11—C12—C13	0.8 (3)
S1—C1—C6—C5	179.80 (16)	C11—C12—C13—C14	0.1 (4)
C2—C1—C6—C7	176.07 (19)	C12—C13—C14—C15	−1.3 (4)
S1—C1—C6—C7	−3.4 (2)	C11—C10—C15—C14	−0.5 (3)
C4—C5—C6—C1	−0.6 (3)	C9—C10—C15—C14	179.5 (2)
C4—C5—C6—C7	−176.9 (2)	C13—C14—C15—C10	1.5 (4)
C8—N1—C7—O1	−2.7 (3)		

### Hydrogen-bond geometry (Å, °)

**Table:**

*D*—H···*A*	*D*—H	H···*A*	*D*···*A*	*D*—H···*A*
C8—H8A···O1^i^	0.99	2.41	3.318 (3)	153
C15—H15···O1^i^	0.95	2.47	3.417 (3)	178

Symmetry codes: (i) −*x*+1/2, *y*−1/2, −*z*+1/2.

## References

[bb1] Ahmad, M., Siddiqui, H. L., Azam, M., Siddiqui, W. A. & Parvez, M. (2009). *Acta Cryst.* E**65**, o2185. 10.1107/S1600536809030773 PMC2970051 21577589

[bb2] Ahmad, M., Siddiqui, H. L., Zia-ur-Rehman, M., Ashiq, M. I. & Tizzard, G. J. (2008). *Acta Cryst.* E**64**, o788. 10.1107/S1600536808007721 PMC2961329 21202281

[bb3] Bernstein, J., Davis, R. E., Shimoni, L. & Chang, N.-L. (1995). *Angew. Chem. Int. Ed. Engl.* **34**, 1555–1573.

[bb4] Blessing, R. H. (1997). *J. Appl. Cryst.* **30**, 421–426.

[bb5] Farrugia, L. J. (1997). *J. Appl. Cryst.* **30**, 565.

[bb6] Hooft, R. (1998). *COLLECT* Nonius B V, Delft, The Netherlands.

[bb7] Kapui, Z., Varga, M., Urban-Szabo, K., Mikus, E., Szabo, T., Szeredi, J., Batori, S., Finance, O. & Aranyi, P. (2003). *J. Pharmacol. Exp. Ther.* **305**, 451–459. 10.1124/jpet.102.044263 12606659

[bb8] Otwinowski, Z. & Minor, W. (1997). *Methods in Enzymology*, Vol. 276, *Macromolecular Crystallography*, Part A, edited by C. W. Carter Jr and R. M. Sweet, pp. 307–326. New York: Academic Press.

[bb9] Sheldrick, G. M. (2008). *Acta Cryst.* A**64**, 112–122. 10.1107/S0108767307043930 18156677

[bb10] Siddiqui, W. A., Ahmad, S., Ullah, I. & Malik, A. (2006). *J. Chem. Soc. Pak.* **28**, 583–589.

[bb11] Singh, S. K., Shivaramakrishna, S., Saibaba, V., Rao, K. S., Ganesh, K. R., Vasudev, R., Kumar, P. P., Babu, J. M., Vyas, K., Rao, Y. K. & Iqbal, J. (2007). *Eur. J. Med. Chem.* **42**, 456–462. 10.1016/j.ejmech.2006.09.021 17097771

[bb12] Vaccarino, A. L., Paul, D., Mukherjee, P. K., de Turco, E. B. R., Marcheselli, V. L., Xu, L., Trudell, M. L., Minguez, J. M., Matia, M. P., Sunkel, C., Alvarez-Builla, J. & Bazan, N. G. (2007). *Bioorg. Med. Chem.* **15**, 2206–2215. 10.1016/j.bmc.2006.07.054 16919959

[bb14] Zia-ur-Rehman, M. Z., Choudary, J. A. amp; Ahmad, S. (2005). *Bull. Korean Chem. Soc* **26**, 1771–1175.

[bb13] Zia-ur-Rehman, M., Choudary, J. A., Elsegood, M. R. J., Siddiqui, H. L. & Khan, K. M. (2009). *Eur. J. Med. Chem.* **44**, 1311–1316. 10.1016/j.ejmech.2008.08.002 18804313

